# Machine learning through cryptographic glasses: combating adversarial attacks by key-based diversified aggregation

**DOI:** 10.1186/s13635-020-00106-x

**Published:** 2020-06-01

**Authors:** Olga Taran, Shideh Rezaeifar, Taras Holotyak, Slava Voloshynovskiy

**Affiliations:** Stochastic Information Processing Group, Department of Computer Science, University of Geneva, 7 Route de Drize, Carouge, GE Switzerland

**Keywords:** Adversarial examples, Defense, Randomization, Diversified aggregation, Black-box attacks, Non-gradient/gradient-based attacks, Machine learning

## Abstract

In recent years, classification techniques based on deep neural networks (DNN) were widely used in many fields such as computer vision, natural language processing, and self-driving cars. However, the vulnerability of the DNN-based classification systems to adversarial attacks questions their usage in many critical applications. Therefore, the development of robust DNN-based classifiers is a critical point for the future deployment of these methods. Not less important issue is understanding of the mechanisms behind this vulnerability. Additionally, it is not completely clear how to link machine learning with cryptography to create an information advantage of the defender over the attacker. In this paper, we propose a key-based diversified aggregation (KDA) mechanism as a defense strategy in a gray- and black-box scenario. KDA assumes that the attacker (i) knows the architecture of classifier and the used defense strategy, (ii) has an access to the training data set, but (iii) does not know a secret key and does not have access to the internal states of the system. The robustness of the system is achieved by a specially designed key-based randomization. The proposed randomization prevents the gradients’ back propagation and restricts the attacker to create a “bypass” system. The randomization is performed simultaneously in several channels. Each channel introduces its own randomization in a special transform domain. The sharing of a secret key between the training and test stages creates an information advantage to the defender. Finally, the aggregation of soft outputs from each channel stabilizes the results and increases the reliability of the final score. The performed experimental evaluation demonstrates a high robustness and universality of the KDA against state-of-the-art gradient-based gray-box transferability attacks and the non-gradient-based black-box attacks (The results reported in this paper have been partially presented in CVPR 2019 (Taran et al., Defending against adversarial attacks by randomized diversification, 2019) & ICIP 2019 (Taran et al., Robustification of deep net classifiers by key-based diversified aggregation with pre-filtering, 2019)).

## Introduction

The advent of deep learning techniques [1] has stimulated the deployment of machine learning in many applications. The DNNs have been applied to solve a wide range of problems in image classification [2, 3], object detection [4, 5], face recognition [6, 7] image caption [8, 9], natural language processing [10, 11], speech recognition [12, 13], drones and robotics [14, 15], malware detection [16, 17], etc., and more science and discovery-related fields, such as drug composition analysis [18], brain circuit reconstruction [19], and DNA mutation impact analysis [20].

Despite the outstanding performance and remarkable achievements, the DNN systems have recently shown to be vulnerable to *adversarial attacks* [21]. These adversarial attacks aim at tricking a decision of the DNN with high confidence during test time by introducing carefully designed perturbations to a chosen target image. These perturbations are usually quite small in magnitude and almost imperceptible to human vision system that makes them almost universal and yet very dangerous. At the same time, such attacks can cause a neural network to produce an erroneous decision about the signal or image. Even worse, the attacked models report high confidence on the produced wrong classification and it is difficult if not impossible to distinguish it from those obtained on the original data. Moreover, the same added perturbation can fool multiple network models with similar or different architectures trained for the same task [22]. Additionally, Kurakin et al. [23] have proven that adversarial examples also exist in physical-world scenarios. This weakness has become a major security concern and seriously questions the usage of the DNN-based systems in many security- and trust-sensitive applications.

The serious implications caused by the adversarial attacks triggered a wide interest of researchers to investigate defenses for deep learning models. In recent years, various defense strategies and countermeasures to protect the DNN against adversarial attacks were proposed [24–26]. However, the growing number of defenses leads to a natural invention of new and even more universal attacks. The diversity of discovered adversarial attacks is quite broad, but without loss of generality, one can cluster all these attacks into three large groups [27, 28]: (1) *white-box* attacks, (2) *gray-box* attacks, and (3) *black-box attacks*. The *white-box* attacks assume that the attacker has a full access to the trained model and training data. Despite a big popularity of this group of attacks, their applicability to real-life systems is questionable due to the fact that most real-world systems do not release their internal configurations and/or trained parameters. The reason behind the usage of this group of attacks is to be compliant with cryptographic principles stating that “a secure system” should assume public knowledge of the algorithm. However, this principle does not completely apply here since the defender does not use any secret key. In fact, both the defender and attacker share the same training data sets. Thus, the defender has no information advantage over the attacker.

The *gray-* and *black-box* scenarios are more suited to real-life applications. The *gray-box* attacks assume that the attacker has certain knowledge about the trained model but there exist some secret unknown elements to the attacker or access to the intermediate results is limited. The *back-box* attack scenario assumes that the attacker only observes the system output to each input without any knowledge about used architecture or possibility to observe the internal states.

**Fig. 1:**
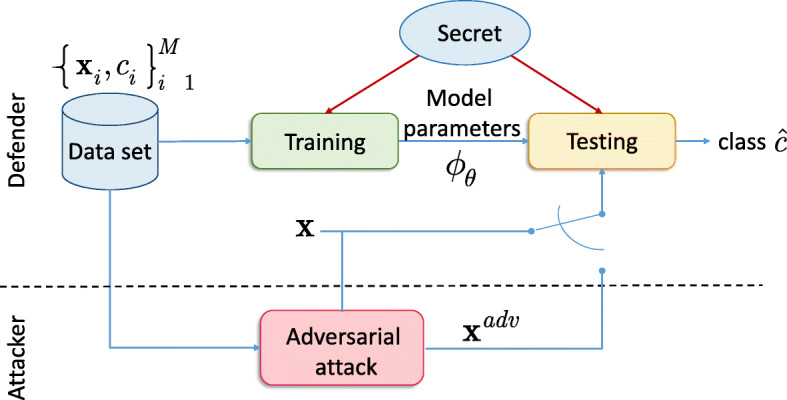
The information access diagram: the defender has an access to the training data and secret shared between the training and test stages while the attacker has only access to the shared training data set

In this paper, we consider an image classification problem and aim at investigating a new family of defense strategies inspired by the second Kerckhoffs’s cryptographic principle [29] that can be applied to both gradient- and non-gradient-based adversarial attacks in *gray-* and *black-box* scenarios. We name it *key-based diversified aggregation* (KDA). The main idea behind the proposed approach is to create an information advantage of the defender over the attacker. The generalized information access diagram of the proposed system is illustrated in Fig. 1. The defender has an access to both training data and secret shared between the training and test stages. We assume that the attacker can only access the training data. The defender can combine the data and the secret in various ways like, for example, by adding secret key-based random noise, by projecting the input onto the random basis vectors generated from the secrete key, via key-driven random cropping or affine transformations, etc. However, since in general case, such perturbations might lead to the classification performance drop, one can create a redundancy by applying these perturbations many times to the input thus creating multi-channel processing. In this way, the classification process is diversified in *L* channels possessing its own regular perturbation. Since the introduced perturbations are known to the defender, the classifier $\phantom {\dot {i}\!}{\phi }_{\boldsymbol {\theta }_{l}}$ in each channel *l*, 1≤*l*≤*L*, is trained only for the certain defender’s perturbation. To reduce a possible negative effect of perturbation that might lead to the information loss in general, the soft outputs of the classifiers in the multi-channel system are aggregated. The final decision is communicated to the output of the system in the form of class label $\hat {c} \in \{1,2,\cdots,M_{c}\}$, where *M*_*c*_ is the number of classes. At the test stage, the defender has both a probe ***x*** and the secret key while the attacker has only the training set. The attacker can produce an adversarial example ***x***^*adv*^ and observe the decision output of system $\hat {c}$ or a rejection. Since the attacker does not have a direct access to the defender’s perturbation that is characterized by a sufficient entropy, the only possibility is to increase the number of adversarial tests to be performed according to the observable output. This makes the adversarial attacks less efficient against this system and more complex.

The proposed method provides the following advantages for the defender over the attacker:

- The use of the secret key creates an information advantage for the defender over the attacker.
- The multi-channel system increases the computational burden of the attacker over the defender. The attacker has to attack at least several channels simultaneously to ensure misclassification outcome.
- The key-based diversification and a limited access to the internal system states do not allow the attacker to build a “bypass” system. Unavailability of the “bypass” system makes it difficult, if not impossible, to use the gradient-based *white-box* attacks, which are more efficient than the “blind” iterative *black-box* attacks.
- The right choice of aggregation operator and a possibility to choose the channels at random provide an additional degree of freedom and increase the security of the whole system.
- Finally, each channel can have an adjustable amount of randomness that allows not only to achieve the required level of defense but it also gives a possibility to adapt to different types of attacks.

**Fig. 2:**
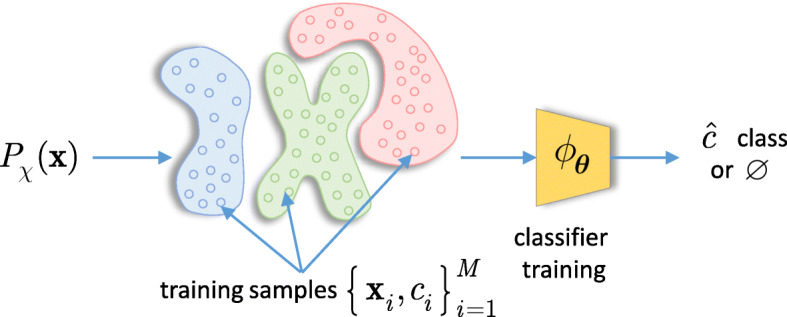
Classifier training: a traditional classifier has an access to training data samples $\{\boldsymbol {\mathrm {x}}_{i}, c_{i}\}_{i=1}^{M}$ generated from *P*_*χ*_(**x**). The classifier learns a set of parameters ***θ*** to output a decision $\hat {c} \in \{1,..., M_{c}\}$ or to reject an input ($\varnothing $)

**Fig. 3:**
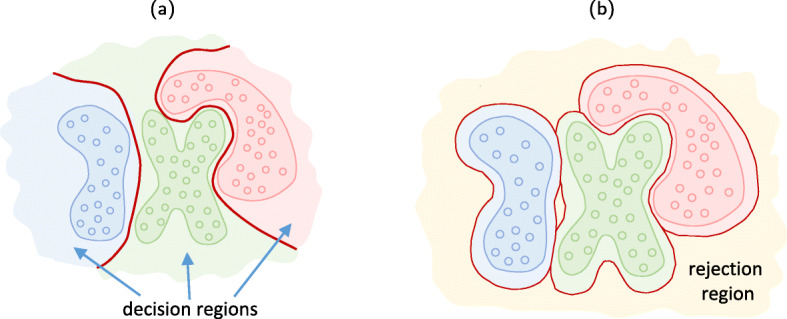
Classifier’s decision boundaries: **a** without rejection and **b** with rejection. Note the difference in the decision regions of trained classifiers

**Fig. 4:**
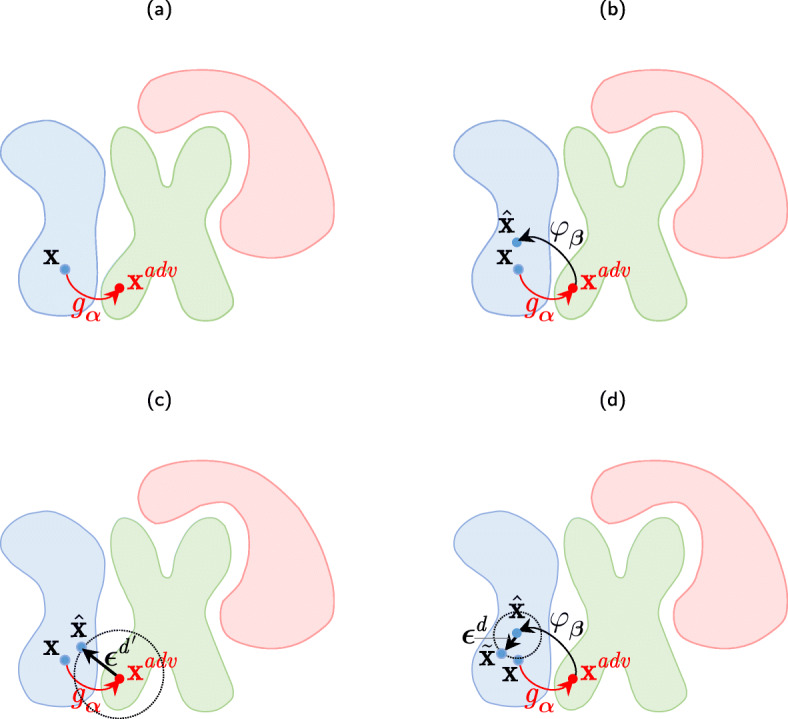
The attacker-defender game in adversarial classification: **a** the attacker produces an adversarial example ***x***^*adv*^ from a host ***x*** by a mapper ***x***^*adv*^=*g*_***α***_(***x***,***ε***); **b** the defender answers by the pre-filtering *φ*_***β***_(***x***^*adv*^) to obtain an estimation $\hat {\boldsymbol {\mathrm {x}}}$ on the original host class manifold; **c** an alternative defense strategy by a randomization of input adversarial image as $\hat {\boldsymbol {\mathrm {x}}} = \boldsymbol {\mathrm {x}}^{adv} + \boldsymbol {\epsilon }^{d^{\prime }}$, the resulting sample will be outside attacker’s target class with a small probability that the resulting sample will be in the original host class that requires the classifiers retraining; **d** the proposed defense strategy consists of pre-filtering by *φ*_***β***_(***x***^*adv*^) and addition of defender’s randomized perturbation ***ε***^*d*^: ***x*** ~=*φ*_***β***_(***x***^*adv*^)+***ε***^*d*^, such that $\lVert \boldsymbol {\epsilon }^{d} \rVert ^{2}_{2} \ll \lVert \boldsymbol {\epsilon }^{d^{\prime }}\rVert ^{2}_{2}$

**Fig. 5:**
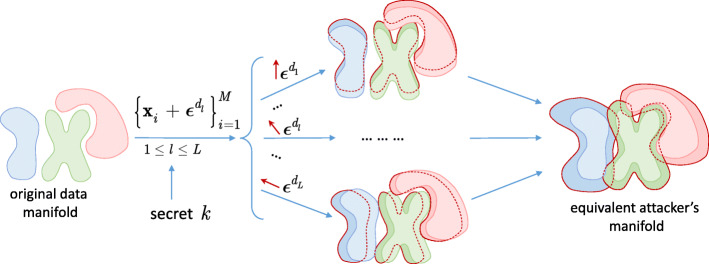
Explanation of multi-channel randomization: given a training data set, the defender introduces a random perturbation $\protect \phantom {\dot {i}\!}{\boldsymbol {\epsilon }}^{d_{l}}$, 1≤*l*≤*L*, to each sample $\{\boldsymbol {\mathrm {x}}_{i}\}^{M}_{i=1}$ and trains *L* classifiers. Since the perturbation is known at training, i.e., all samples obtain the stationary “bias” by $\protect \phantom {\dot {i}\!}{\boldsymbol {\epsilon }}^{d_{l}}$ for the same classifier *l*, the randomization has a limited impact on the classifier performance. Since the attacker has no access to the defender’s perturbations and all of them are equilikely, an equivalent manifold for the attacker is expanded thus leading to higher entropy and thus increasing the learning complexity

The present paper is a further extension of our previous framework proposed in [30, 31]. In particular, we extend and explain in more details the main elements and features of the proposed protection mechanism reflected in Figs. 1, 2, 3, 4, and 5. Additionally, new extended experiments have been performed to demonstrate the following:

- The robustness of the proposed multi-channel system in face of transferability attacks in *gray-box* scenario against new PGD attack [32]. The obtained results are given in Table 2.
- The transferability of the attacks with respect to the vanilla classifiers. The results are presented in Tables 1 and 2. In general, we show that the considered attacks do not possess the high transferability from a single-channel model to a single-channel model. Moreover, we confirm that the transferability to the proposed multi-channel model with KDA is even weaker.

**Table 1:** Classification error (%) on the first 1000 test samples for the *gray-box* C&W transferability attacks from a single-channel model to a multi-channel model

Data type	Attacked	Transferability	Transferability KDA		
	vanilla	vanilla	# channels · # classifiers		
			3	6	9
*MNIST*					
Original	1	0.9	0.5	0.5	0.5
*C&W ℓ*_2_	100	6.69	4.69	4.81	4.02
*C&Wℓ*_0_	100	14.2	7.27	7.51	6.78
*C&Wℓ*_*∞*_	99.99	4.77	2.73	2.28	2.08
*Fashion-MNIST*					
Original	7.5	7.5	8.1	7.4	7.6
*C&Wℓ*_2_	100	11.2	9.26	8.68	8.9
*C&Wℓ*_0_	100	11.82	10.41	9.97	10
*C&Wℓ*_*∞*_	99.9	11.59	9.19	8.52	8.79
*CIFAR-10*					
Original	21	20.6	21.2	19.6	19.5
*C&Wℓ*_2_	100	25.09	22.42	21.3	21.04
*C&Wℓ*_0_	100	30.71	24.58	23.52	23.03
*C&Wℓ*_*∞*_	100	25.42	22.8	21.39	21.21

**Table 2:** Classification error (%) on the first 1000 test samples (CIFAR-10) for the *gray-box* PGD transferability attacks from a single-channel model to a multi-channel model with randomly selected channels (the average results over 10 runs)

Data type	Attacked	Transferability	Transferability KDA			
	vanilla	vanilla	# channels · # classifiers			
			3	5	7	9
*VGG 16*						
Original	10.7	11.7	11.6	9.9	9.5	9
PGD	16.1	15.2	14.25	12.16	11.75	11
*ResNet 18*						
Original	9.5	10.6	11.7	9.3	8.8	8.1
PGD	17.9	14.9	14.7	11.29	10.67	9.7
- The transferability of the attacks from one multi-channel system to another multi-channel system assuming that the attacker has full knowledge about the classification model architecture and used defense mechanism except the secret keys of defender. The results are given in Table 3 and show the high robustness of proposed system to such kind of attacks.

**Table 3:** Classification error (%) on the first 1000 test samples (CIFAR-10) for the *gray-box* OnePixel transferability attacks from a multi-channel model to a multi-channel model under different keys

Data type	KDA with different keys		
	# channels · # classifiers		
	3	6	9
VGG16			
Original	12.6	11.2	10.5
OnePixel *p*=1	13.07	10.98	10.4
OnePixel *p*=3	12.72	11.37	10.3
OnePixel *p*=5	12.6	11.35	10.8
ResNet18			
Original	9.95	8.4	7.7
OnePixel *p*=1	9.75	8.17	7.8
OnePixel *p*=3	10	8.35	7.8
OnePixel *p*=5	10.38	8.39	8.1

**Table 4:** Classification error (%) on the first 1000 CIFAR-10 test samples for the direct *black-box* OnePixel attacks

Data type	Attacked	Attacked KDA		
	vanilla	# channels · # classifiers		
		3	6	9
VGG16				
Original	10.7	11	9.2	8.9
OnePixel *p*=1	58.04	11	9.5	8.7
OnePixel *p*=3	72.13	10.9	8.9	8.3
OnePixel *p*=5	79.02	12.1	9.3	9.1
ResNet18				
Original	9.5	11.1	9.1	7.8
OnePixel *p*=1	36.96	11.5	9	7.7
OnePixel *p*=3	49.85	11.5	9.1	7.8
OnePixel *p*=5	59.74	11.7	9.2	7.8
- Finally, the system performance with the randomized aggregation of multi-channel outputs according to Fig. 6 is investigated. The corresponding results are presented in Tables 2, 5, and 6 and demonstrate that the key-based aggregation can be used as an extra layer of protection.

**Fig. 6:**
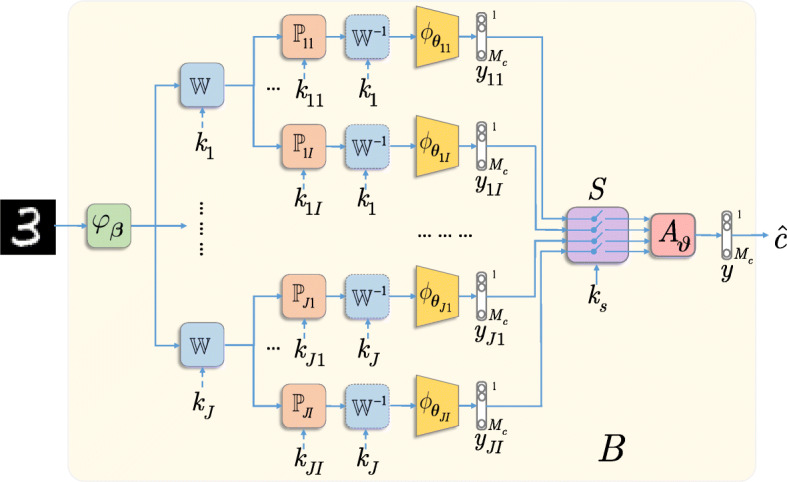
Generalized diagram of the proposed multi-channel system with the KDA

**Table 5:** Classification error (%) on the first 1000 test samples for the *gray-box* C&W transferability attacks from a single-channel model to a multi-channel model with randomly selected channels (the average results over 10 runs)

Data type	Transferability KDA		
	# channels · # classifiers		
	3	5	7
*MNIST*			
Original	0.6	0.5	0.6
*C&Wℓ*_2_	5.06	4.77	4.44
*C&Wℓ*_0_	7.77	7.3	7.12
*C&Wℓ*_*∞*_	3.41	3.12	2.77
*Fashion-MNIST*			
Original	8.2	8.2	8.1
*C&Wℓ*_2_	9.4	9.1	8.84
*C&Wℓ*_0_	10.58	10.52	10.27
*C&Wℓ*_*∞*_	9.33	9.09	8.83
*CIFAR-10*			
Original	21.2	20.5	19.9
*C&Wℓ*_2_	22.92	21.22	21.1
*C&Wℓ*_0_	27.82	25.46	24.33
*C&Wℓ*_*∞*_	24.57	22.33	21.86

**Table 6:** Classification error (%) on the first 1000 test samples (CIFAR-10) for the multi-channel system against the direct *black-box* OnePixel attacks with randomly selected channels (the average results over 10 runs)

Data type	Attacked KDA		
	# channels · # classifiers		
	3	5	7
VGG16			
Original	11.7	9.5	9.3
OnePixel *p*=1	11.3	9.6	9
OnePixel *p*=3	11.5	9.8	8.9
OnePixel *p*=5	12	10.6	9.4
ResNet18			
Original	11.1	9.7	8.8
OnePixel *p*=1	11.1	9.2	8.9
OnePixel *p*=3	11.4	9.6	8.8
OnePixel *p*=5	10.9	9.8	9.1

**Notations.** We use small bold letters ***x*** to denote a signal that can be represented in 1D, 2D, or 3D format. *ϕ*_***θ***_ denotes the classifier model with the parameters ***θ***, *c* is used to denote the class label, and ***y*** is a soft output of the classifier *ϕ*_***θ***_, where *c* corresponds to the maximum value in ***y***, ***ε*** corresponds to the adversarial perturbation, and ***ε***^*d*^ means the defender’s perturbation.

## Previous work: defenses and attacks

The diagram shown in Fig. 2 illustrates a traditional view on the classification process. Assume that the data samples are drawn from a distribution *P*_*χ*_(***x***) possessing *M*_*c*_ classes. The labeled training samples represent the training data set $\{\boldsymbol {\mathrm {x}}_{i}, c_{i}\}_{i=1}^{M}$ with *M* training samples. At the training stage, the classifier *ϕ*_***θ***_ uses the available training data to learn the parameters ***θ***. At the test stage, given a test sample ***x***, the trained classifier *ϕ*_***θ***_ outputs one of the classes $\hat {c} \in \{1,2,\cdots,M_{c}\}$. A rejection option can be also naturally envisioned. The trained decision boundaries are schematically illustrated in Fig. 3.

Since Kurakin et al. [23] demonstrated the vulnerability of the DNN to adversarial attacks, one can observe an increasing interest to the investigation of both new attacks and development of efficient countermeasures.

First, we consider a generic attack on the above classifier. As shown in Fig. 4a, the attacker produces an adversarial example ***x***^*adv*^ from a host sample ***x*** of a class *c* by a mapper *g*_***α***_: ***x***^*adv*^=*g*_***α***_(***x***,***ε***) with some perturbation ***ε*** in such a way to fool the classifier *ϕ*_***θ***_: *ϕ*_***θ***_(***x***^*adv*^)=*c*^*adv*^, i.e., to force the classifier to produce an output *c*^*adv*^≠*c*. Generally, *g*_***α***_ can be any non-linear mapper. However, a simple additive attack has become the most popular one: ***x***^*adv*^=***x***+***ε***. The classic approach assumes that the attacker has an access to the same training data samples as the defender. Thus, there is no information advantage of the defender over the attacker. Moreover, having general knowledge about the used classifier architecture, cost function, and training algorithm, the attacker can learn with a certain degree of precision the same decision boundaries as the defender (Fig. 3).

### Defense strategies

Nowadays, different types of defense strategies have been developed [26]. Without pretending to be exhaustive in our overview, we only mention some of the well-known families of defense strategies.

Probably, the largest family of defense strategies is based on *retraining*. Most successful works in this direction are *network distillation* proposed by Papernot et al. in [33] and *adversarial retraining* investigated by Goodfellow et al. [34], Kurakin et al. [23], Wu et al. [35], etc. The main reason for this interest is based on a belief that a well-trained classifier that has access to the adversarial examples can adjust its decision boundaries and efficiently filter them out. However, the attacker has always the last word in this game and can create new unseen types of adversarial examples. At the same time, to envision all possible adversarial examples on the side of the defender or to generate them, it looks practically infeasible. It should be pointed out that in this setting, the defender has no information advantage over the attacker.

The second large family of defense strategies is based on a *detection*-*rejection* approach. If one assumes that the adversarial examples are based on a modification of original data, it is natural to expect that this adversarial modification leads to the difference in statistics of original data and adversarial ones. It is worth mentioning that the adversarial example detection is similar in nature to a steganalysis problem, where the digital watermarking community has developed a rich family of methods. Summarizing this experience, one can mention that to train efficient detectors of adversarial attacks, it is needed to either know a model describing an adversarial modulation along with the statistics of original data [36] or have an access to the training data sets of original and adversarial examples. Some examples of these strategies include [37–40]. The detection of adversarial attacks might work, if the attack statistics remain the same. Unfortunately, the detection of new attacks requires re-training and there is no guarantee that unseen examples are detectable. Finally, similarly to steganography, advanced attackers will mask the statistics of adversarial perturbations by host statistics as it is done in smooth adversarial attack [41]. This makes the tasks of defender very difficult due to low distinguishability of host and perturbation statistics.

Alternatively, one can envision an active defense strategy when the defender attempts at removing or decreasing the effect of adversarial perturbation by *pre-processingφ*_***β***_ via different types of filtering to bring the input to the original data manifold as shown in Fig. 4b. Similar strategy was very efficient against robust digital watermarking, where the watermark was considered as an additive noise and the pre-filtering removed this watermark by denoising. Since the denoising is known to be very efficient in the flat regions [42] to destroy the remaining watermark completely, the additive noise was added to the regions of textures and edges. The goal of filtering can be achieved in several ways. If the model of adversarial modulation is known, the defender can develop an efficient filtering strategy using an analytically derived filter when the model of the image is assumed to be known too. Otherwise, a machine learnable image model can be used. If the model of adversarial perturbation is unknown but the training samples of original data and its adversarial perturbations are available, one can design a network mapping the adversarial input to the clean data. Finally, when only the original data are available, one can train an auto-encoder on it and then apply it to the adversarial data. The trained decoder will attempt at generating almost clean output by projecting an adversarial example onto the manifold of training data encoded into a structure of the auto-encoder. We will refer to this form of filtering as *regeneration*. For example, Gu et al. in [43] propose a deep contractive auto-encoder that is a variant of the classical auto-encoder with an additional penalty increasing the robustness to adversarial examples. Meng et al. in [44] introduce *MagNet*, which combines the detector and regeneration networks. The filtering via denoising was considered in [44–46] and via compression in [47, 48]. However, since, in general case, the pre-processing *φ*_***β***_ is deterministic in nature, sooner or later, the attacker can learn and bypass it.

This leads to the need to use *randomization* as a second step [42]. Generally, the idea behind the randomization can be considered as a perturbation of adversarial image with distortion $\boldsymbol {\epsilon }^{d^{\prime }}$ defined by the defender as shown in Fig. 4c. The resulting sample is expected to be outside the attacker’s target class. In practice, the randomization is considered in various ways and might include (a) the randomization of input, (b) the randomization of feature vectors, (c) the randomization of filters, and (d) the randomization of any decision-making function parameters. For example, in [25] the authors propose to apply a random permutation to the input data as a form of randomization. Another direction is to randomize the input data by adding noise [49–51]. The input image randomization via random image resizing and padding is investigated in [52]. In [53], the authors explore the idea of stochastically combining different image transforms like, for example, discrete Fourier transform (DFT) domain perturbation, color changing, noise injection, and zooming into a single barrage of randomized transformations to build a strong defense. The idea of DNN feature randomization is examined in [54–56]. Since a particular form of randomization is unknown to the attacker, the defender gains an important information advantage over the attacker. However, it can be achieved only under the condition that the applied defender’s randomization tricks are properly incorporated into the classifier. Otherwise, an uninformed classifier will treat them as noise or degradation that unavoidably leads to the drop in the classification accuracy. Moreover, as it was noticed by all authors of the above papers, another problem of randomization techniques is related to the fact that after randomization, the inputs might be randomly swapped between the classes, which leads to the drop in the classification accuracy too.

In Fig. 4d, the proposed idea of combining the pre-processing and randomization techniques is explained. First of all, it includes returning the input sample to the original data manifold through an appropriate pre-filtering and, secondly, to make the defense stochastic and to create the information advantage for the defender, the perturbing the image with a distortion ***ε***^*d*^, such that $\lVert \boldsymbol {\epsilon }^{d} \rVert ^{2}_{2} \ll \lVert \boldsymbol {\epsilon }^{d^{\prime }}\rVert ^{2}_{2}$. However, in the case of the complex geometry of classes and strong adversarial attacks, the strong perturbation ***ε***^*d*^ could be required too, and as a consequence, the input sample swapping between the classes could not be excluded. To overcome this shortcoming, in this paper, we propose a multi-channel randomization technique as shown in Fig. 5. The main idea is that in each channel *l*, 1≤*l*≤*L*, the defender introduces a random perturbation $\phantom {\dot {i}\!}\boldsymbol {\epsilon }^{d_{l}}$ to each sample $\{\boldsymbol {\mathrm {x}}_{i}\}^{M}_{i=1}$ and trains *l*^th^ classifier. Since the perturbation is known at training, i.e., all samples obtain a stationary “bias” by $\phantom {\dot {i}\!}\boldsymbol {\epsilon }^{d_{l}}$ for the same classifier *l*, the randomization has a limited impact on the classifier performance: all classes’ manifolds will be just moved along the direction of perturbation on $\phantom {\dot {i}\!}\boldsymbol {\epsilon }^{d_{l}}$, and if they are separable in the original space, then they will stay separable in a new space as well. This allows to avoid the decrease of classification accuracy. Moreover, in this case, the perturbation $\phantom {\dot {i}\!}\boldsymbol {\epsilon }^{d_{l}}$ might be sufficiently big to face strong attacks. From the point of the attacker, since he has no access to the defender’s perturbations $\phantom {\dot {i}\!}\boldsymbol {\epsilon }^{d_{l}}$, all of them are equilikely and an equivalent manifold for the attacker expands thus leading to higher entropy and increases the attacker’s learning complexity. Moreover, the targeted attacks become more difficult since the boundaries between the classes on the expanded manifold are not clearly defined due to the random perturbations $\phantom {\dot {i}\!}\boldsymbol {\epsilon }^{d_{l}}$.

### Adversarial attacks

Without loss of generality, one can group the state-of-the-art adversarial attacks against the DNN classifiers into two main groups [26]:

1. *Gradient*-based attacks. The core idea behind this group of attacks consists of the back propagation of the targeted class label to the input layer. A function of the gradient is considered as an adversarial noise that is added to a host image. Obviously, to successfully propagate the gradient via a network, it should be end-to-end differentiable.
  Without pretending to be exhaustive in our overview, we would like to mention some well-known attack strategies of this group. The L-BFGS attack proposed by Szegedy et. al. in [57] is time-consuming due to the used expensive linear search and, as a consequence, is impractical for real-life applications. However, this attack served as a basis for several more successful attacks such as *Fast Gradient Sign Method* (FGSM) [34]. In contrast to L-BFGS, FGSM is fast but not all the time gives the minimal adversarial perturbation between original and targeted samples. FGSM method has several successful extensions, like FGSM with momentum [58], *One-step Target Class Method* (OTCM) [23], RAND-FGSM algorithm [59], proposed in [23] *Basic Iterative Method* (BIM), projected gradient descent (PGD) [32] the generalized version of BIM, and *Iterative Least-Likely Class Method* (ILLC). In addition, it should also be mentioned the *Jacobian-based Saliency Map Attack* (JSMA) [60] and the *DeepFool* approach [61] with its extension *Universal perturbation* [62]. Moreover, one should note the attack proposed by Carlini and Wagner in [63] that we will refer to as C&W attack. As it has been shown in many works, like for example in [64] and [65], this attack is among the most efficient ones against many existing defense mechanisms. Finally, Athalye et. al. in [66] propose Backward Pass Differentiable Approximation technique that aims at avoiding the gradient masking in *white-box* scenario.
2. *Non-gradient*-based attacks
  The attacks of this group do not require any knowledge of the DNN gradients or the need of network differentiability. The most well-known members of this group are the *Zeroth Order Optimization* (ZOO) [67] and the *OnePixel Attack* [68].

In our work, we will consider the most successful representatives of each group, namely, gradient-based C&W attack and non-gradient-based OnePixel attack.

In general case, for an input image $\boldsymbol {\mathrm {x}} \in \mathbb {R}^{N \times S}$ with a class label *c*∈{1,2,⋯,*M*_*c*_}, the optimization problem of finding an adversarial example with the additive perturbation ***x***^*adv*^=***x***+***ε*** and target class *c*^*adv*^ can be formulated as follows:

1 $$ \begin{array}{cl} \min_{\boldsymbol{\epsilon}} & \mathcal{L} \Big (c^{adv}, \phi_{\boldsymbol\theta}(\boldsymbol{\mathrm{x}} + \boldsymbol{\epsilon}) \Big) + \lambda \| \boldsymbol{\epsilon} \|_{p}, \\ s.t. & \boldsymbol{\mathrm{x}} + \boldsymbol{\epsilon} \in [0,1]^{N \times S}, \end{array}   $$

where $\mathcal {L}(.)$ is a classification loss, *ϕ*_***θ***_ is a targeted classifier, *c*≠*c*^*adv*^, *λ* is a Lagrangian multiplier, and *ℓ*_*p*_-norm is defined as:

$$\begin{array}{*{20}l} \begin{array}{cl} \|\boldsymbol{\epsilon}\|_{p} & = \Bigg (\sum_{i=1}^{N \times S} |\epsilon_i|^{p} \Bigg)^{\frac{1}{p}}, \\ \text{with} & 0 \le p \le 2. \end{array} \end{array} $$

#### C&W attack

The C&W attack proposed by Carlini and Wagner in [63] is among the most efficient attacks against many reported so far defense strategies. The authors find the formulation (1) difficult for solving directly due to the high non-linearity and propose an alternative definition:

2 $$ \begin{array}{cl} \min_{\boldsymbol{\epsilon}} & a \cdot f(\boldsymbol{\mathrm{x}} + \boldsymbol{\epsilon}) + \| \boldsymbol{\epsilon} \|_{p}, \\ s.t. & \boldsymbol{\mathrm{x}} + \boldsymbol{\epsilon} \in [0,1]^{N \times S}, \end{array}  $$

where *a*>0 is a suitably chosen constant, *f*(.) is a new objective function such that *ϕ*_***θ***_(***x***+***ε***)=*c*^*adv*^, if and only if *f*(***x***+***ε***)≤0. In [63] the authors investigate several objective functions *f*(.), and as the most efficient one, they propose:

3 $$ {}f(\boldsymbol{\mathrm{x}}^{adv}) = \max \left(\max_{l \ne c^{adv}} \left(Z\left(\boldsymbol{\mathrm{x}}^{adv}\right)_{l} \right) - Z\left(\boldsymbol{\mathrm{x}}^{adv}\right)_{c^{adv}}, -\kappa \right),  $$

where *l* is an index of any class while *c*^*adv*^ is an index of the adversarial class; $Z(\boldsymbol {\mathrm {x}}) = \phi _{\boldsymbol {\theta }^{n-1}}(\boldsymbol {\mathrm {x}})$ is the result of the network *ϕ*_***θ***_ before the last activation function that, in case of classification, usually it is a *softmax*; and *κ* is a constant that controls the confidence of the attack.

#### PGD attack

Additionally to the C&W attack, the PGD attack [32] that is an iterative version of FGSM attack and a generalized version of BIM attack was considered. The PGD solves the optimization problem (1) by computing an adversarial example at the iteration *t*+1 as:

4 $$ \begin{array}{cl} \boldsymbol{\mathrm{x}}^{adv}_{t+1} = Proj \bigg(\boldsymbol{\mathrm{x}}^{adv}_{t} + \alpha \cdot sign \Big(\nabla{\scriptsize{\boldsymbol{\mathrm{x}}}}\mathcal{L}\left(c^{adv}, \phi_{\boldsymbol{\theta}}(\boldsymbol{\mathrm{x}}^{adv}_{t}) \right)\Big)\bigg) \end{array}  $$

where *Proj*(.) keeps $\boldsymbol {\mathrm {x}}^{adv}_{t+1}$ within a predefined perturbation range and valid image range and *α* a is the magnitude of the adversarial perturbation in each iteration.

#### OnePixel attack

OnePixel attack was proposed by Su et al. in [68]. This attack uses a Differential Evolution (DE) optimization algorithm [69] for the attack generation. The DE algorithm does not require the objective function to be differentiable or known, but instead, it observes the output of the classifier as a black-box output. The OnePixel attack aims at perturbing a limited number of pixels in the input image $\boldsymbol {\mathrm {x}} \in \mathbb {R}^{N \times S}$. The optimization problem is formulated as:

5 $$ \begin{array}{cl} \min_{\boldsymbol{\epsilon}} & \mathcal{L}\left(c^{adv}, \phi_{\boldsymbol{\theta}}(\boldsymbol{\mathrm{x}} + \boldsymbol{\epsilon})\right),\\ s.t. & \|\boldsymbol{\epsilon}\|_{0} \le d, \\ \end{array}  $$

where *d* is a number of pixels to be modified in the original image ***x*** and $\mathcal {L}(.)$ is a classification loss.

## Classification algorithm based on KDA

The generalized diagram of the proposed multi-channel system with the KDA is shown in Fig. 6. It consists of six main building blocks:

1. *Pre-filteringφ*_***β***_(***x***) that has an optional character. The goal of this block is to return the input image ***x*** back to the manifold of the original class by removing high-magnitude outliers introduced by the attacker, if any. One can choose a broad range of pre-filtering algorithms from a simple local mean filter to more complex algorithms such as BM3D [70] or based on DNN mappers [71].
2. *Pre-processing* of the input data in a *transform domain* via a mapping $\mathbb {W}_{j}$, 1≤*j*≤*J*. In general, the transform $\mathbb {W}_{j}$ can be any linear data-independent mapper. For example, it can be a random projection with the dimensionality reduction or expansion, or belong to a family of orthonormal transformations ($\mathbb {W}_{j}\mathbb {W}^{T}_{j} = \mathbb {I}$) like DFT (discrete Fourier transform), DCT (discrete cosines transform), and DWT (discrete wavelet transform). Moreover, $\mathbb {W}_{j}$ can also be a learnable transform. However, it should be pointed out that from the point of view of the robustness to adversarial attacks, the data-independent transform $\mathbb {W}_{j}$ is of preference to avoid any leakage about it from the training data. Furthermore, $\mathbb {W}_{j}$ can be based on a secret key *k*_*j*_.
3. *Data-independent processing*$\mathbb {P}_{ji}$, 1≤*i*≤*I* presents the randomization part and serves as a defense against gradient back propagation to the direct domain and the manifold expanding. One can envision several cases. As shown in Fig. 7a, $\mathbb {P}_{ji} \in \{0, 1\}^{l \times n}$, *l*<*n*, presents a lossy sampling of the input image of length *n*, as considered in [72]. In Fig. 7b, $\mathbb {P}_{ji} \in \{0, 1\}^{n \times n}$ is a lossless permutation, similar to [25]. Finally, in Fig. 7c, $\mathbb {P}_{ji} \in \{-1, 0, +1\}^{n \times n}$ corresponds to sub-block sign flipping. The yellow color highlights the key defined region of key-based sign flipping. This operation is reversible and thus lossless for an authorized party. Moreover, to make the *data-independent processing* irreversible for the attacker, it is preferable to use a $\mathbb {P}_{ji}$ based on secret key *k*_*ji*_.

**Fig. 7:**
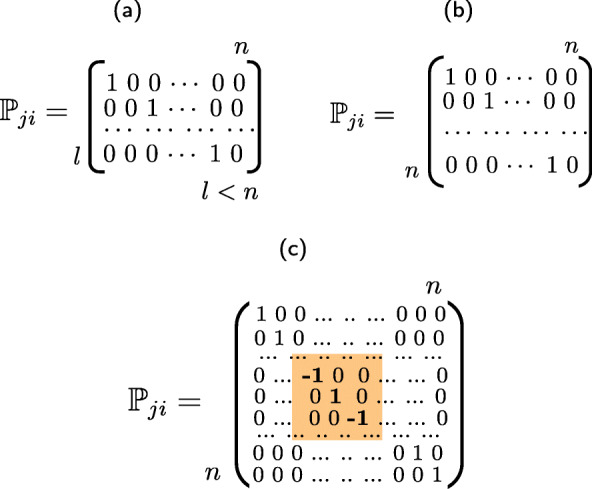
Randomized transformation $\mathbb {P}_{ji}$, 1≤*j*≤*J*, 1≤*i*≤*I* examples: **a** randomized sampling, **b** randomized permutation, and **c** randomized sign flipping in the sub-block defined in orange. All transforms are key-based
4. *Classification block*$\phantom {\dot {i}\!}\phi _{\boldsymbol {\theta }_{ij}}$ can be represented by any family of classifiers. However, if the classifier is designed for classification of data in the direct domain, then it is preferable that it is preceded by $\mathbb {W}_{j}^{-1}$. This concerns the usage of convolutional or fully connected layers.
5. *Classifiers’ selectionS* with a key *k*_*s*_ randomly selects *J*_*s*_ classifiers’ outputs out of *JI* pre-trained classifiers’ outputs for a further aggregation.
6. *Aggregation blockA*_***𝜗***_ can be represented by any operation ranging from a simple summation to learnable operators adapted to the data or to a particular adversarial attack.

As it can be seen in Fig. 6, the chain of processing represented by the blocks 2, 3, and 4 can be organized in a parallel multi-channel structure that is followed by the classifiers’ selector and the *aggregation block*. The final decision about the class is made based on the aggregated result. The rejection option can be also envisioned.

It should be pointed out that the access to the intermediate results inside the considered system provides the attacker a possibility to use the full system as a *white-box*. The attacker can discover the secret keys *k*_*j*_ and/or *k*_*ji*_, and make the system end-to-end differentiable using the Backward Pass Differentiable Approximation technique [66] or via replacing the key-based blocks by the “bypass” mappers. Therefore, it is important to restrict the access of the attacker to the intermediate results within the block *B* (see Fig. 6). That satisfies our assumption about *gray-* and *black-box* attacks. Additionally, it is in the accordance with Kerckhoffs’s cryptographic principle when we assume that the algorithm and architecture are known to the attacker besides the used secret key that in our case corresponds to the secret perturbations.

The training of the described classification architecture can be performed according to:

6 $$ \begin{aligned} ({\hat{\vartheta}}, \{{\hat{\theta}_{ji}} \}) = \underset{{\vartheta}, \{{\theta}_{ji}\}}{\text{argmin}} \sum_{t=1}^{T} \sum_{j=1}^{J} \sum_{i=1}^{I} \mathcal{L}(c_{t}, A_{{\vartheta}}(\phi_{{{\theta}}_{ji}}(\mathbb{W}_{j}^{-1} \mathbb{P}_{ji}\mathbb{W}_{j} \; \varphi_{{\beta}}({\mathrm{x}}_{t})))), \\ \end{aligned}  $$

where $\mathcal {L}$ is a classification loss, *c*_*t*_ is a class label of the sample ***x***_*t*_, *A*_***𝜗***_ corresponds to the aggregation operator with parameters ***𝜗***, *T* equals to the number of training samples, *J* is the total number of channels, and *I* equals to the number of classifiers per channel that, in general, can be different for each channel *j*. For practical implementation, we will keep *I* equal for all channels. $\phi _{\boldsymbol \theta _{ji}}$ is the *i*th classifier of the *j*th channel, and ***θ*** denotes the parameters of the classifier.

In the proposed system, we will consider several practical simplifications leading to information and complexity advantages for the defender over the attacker:

- The defender training is performed per channel independently up to *selection* and *aggregation* blocks. Since *J*_*s*_ classifiers’ outputs out of *JI* pre-trained classifiers are chosen for the aggregation by the defender at the test stage, the attacker should target to attack a subset of classifiers to trick the final decision. To guess a potentially chosen subset, the attacker faces $\binom {JI}{J_{s}}$-combinatorial problem that under properly chosen *JI* and *J*_*s*_ can represent a considerable complexity burden for the attacker. At the same time, the attacker cannot introduce a single perturbation to trick all classifiers simultaneously.
- The blocks of *data-independent processing*$\mathbb {P}_{ji}$ aim at preventing gradient back propagation into the direct domain, but the classifier training is adapted to a particular $\mathbb {P}_{ji}$ in each channel.
- It will be shown further by the numerical results that the usage of the multi-channel architecture with the following aggregation stabilizes the results’ deviation due to the use of randomization or lossy transformations $\mathbb {P}_{ji}$, if such are used.
- The right choice of the *aggregation* operator *A*_***𝜗***_ provides an additional degree of freedom and increases the security of the system through the possibility to adapt to specific types of attacks.
  In general case, as an aggregation operator, the defender could use the following:
  - An additional classification network that takes as an input the soft outputs of multi-channel classifiers and outputs the final prediction. These multi-channel outputs could be, for example, aggregated into a 1D vector via summation, concatenation, etc;
  - The majority voting of the multi-channel outputs;
  - The summation of the multi-channel outputs with the maximum class selection.
  Generally speaking, since the multi-channel classifier could be trained independently from the aggregation block, the choice of aggregation operator could be defined experimentally.
- Moreover, the overall security level considerably increases due to the independent randomization in each channel. The main advantage of the multi-channel system consists in the fact that each channel can have an adjustable amount of randomness that allows to obtain the required level of defense against the attacks. In a one-channel system, the amount of introduced randomness can be either insufficient to prevent the attacks or too high that leads to a drop in classification accuracy. Therefore, having a channel-wise distributed randomness is more flexible and efficient for the above trade-off.

It should be pointed out that the overall complexity of training the multi-channel system is *I*×*J* higher compared to a single-channel system. At the same time, one should note that the aggregation allows relaxing a network fine-tuning complexity. Thus, there is no need in expensive parameters fine-tuning in a multi-channel system in contrast to a single-channel counterpart. The channel aggregation allows to achieve an equivalent performance with “weakly” trained classifiers with lower overall complexity. For the final training, the multi-channel classifiers had to have only different secret keys and different starting initialization per channel. Moreover, in case of non-deep aggregation strategies, the defender training could be performed independently up to *selection* and *aggregation* blocks. This fact allows the defender to train several channels in parallel.

## Randomization using key-based sign flipping in the DCT domain

One of the defense’s core elements in the proposed multi-channel architecture shown in Fig. 6 is the input image randomized diversification via data-independent processing $\mathbb {P}$. The simplest case of such a diversification can be considered for the direct domain with the permutation of input pixels. In fact, the algorithm proposed in [25] reflects this idea for a single channel. However, despite the reported efficiency, a single-channel architecture is subject to a drop in classification accuracy, even for the original, i.e., non-adversarial, data. The performance of a permutation-based defense in a multi-channel setting has been investigated in [30]. The obtained results demonstrate a high sensitivity to the gradient perturbations that degrades the performance of the classifiers. It has been shown in [30, 31] that the preservation of local correlation helps preserve the loss of the gradients and drop of classification accuracy.

**Fig. 8:**
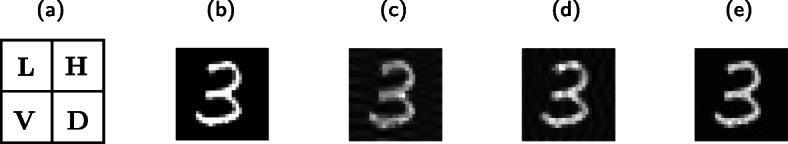
Local key-based sign flipping in the DCT sub-bands: **a** sub-bands, **b** original image, **c** image with a sign flipping in V sub-band, **d** image with a sign flipping in H sub-band, and **e** image with a sign flipping in D sub-band

In this paper, we use the DCT as the operator $\mathbb {W}$ and the local sign flipping $\mathbb {P}_{ji} \in \{-1, 0, 1\}^{n \times n}$ based on the individual secret key *k*_*ji*_ for each classifier *ϕ*_***θ***_. The term *local* means that the processing is done only in some sub-band or block of the input image. The length of the secret key *k*_*ji*_ equals the length of the corresponding sub-band, i.e., *n*×*n*. In general, the image can be split into overlapping or non-overlapping sub-bands of different sizes and different positions that are kept in secret. In our experiments for the simplicity and interpretability, we split the image in the DCT domain into four non-overlapping fixed sub-bands of the same size denoted as follows: (*L*) top left that represents the low frequencies of the image, (*V*) vertical, (*H*) horizontal, and (*D*) diagonal sub-bands as illustrated in Fig. 8a. The key-based sign flipping is applied independently in *V*, *H*, and *D* sub-bands keeping all other sub-bands unchanged. The length of secret key in each sub-band corresponds to *n*×*n*=image size/2×image size/2. The effects of such processing after the inverse DCT transform are perceptually almost unnoticeable and exemplified in Fig. 8c–e.

**Fig. 9:**
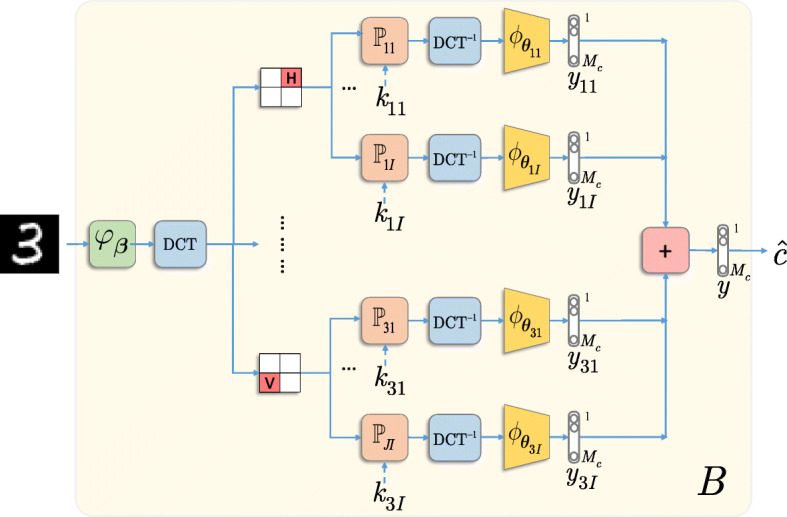
Multi-channel classification with the local DCT sign flipping

The corresponding multi-channel architecture is illustrated in Fig. 9. For simplicity, as an aggregation operator *A*_***𝜗***_, we use a simple summation and the selector *S* uses the outputs of all classifiers *JI*. For the pre-filtering *φ*_***β***_, we use a custom filter based on a difference of the point of interest in the center of the window with the median value in the window of size 3×3 around this point. If the magnitude of difference exceeds a specified threshold, the pixel is considered to be corrupted by the adversary and its value is replaced by a mean value computed in the window, or otherwise, it is kept intact. Finally, under the introduced perturbation, each classifier $\phantom {\dot {i}\!}\phi _{{\boldsymbol \theta }_{ji}}$ is trained independently as:

7 $$ \hat{\theta}_{ji} = \underset{{\theta_{ji}}}{\text{argmin}} \sum_{t=1}^{T} \mathcal{L}(c_{t}, \phi_{{\theta_{ji}}}(\mathbb{W}^{-1}\mathbb{P}_{ji}\mathbb{W}\,  {\varphi}_{\beta}({\textrm{x}}_{t}))) .    $$

The soft outputs of trained classifiers are aggregated by the summation as shown in Fig. 9.

## Results and discussion

### Attacks’ scenarios

Accordingly to the central concept of the proposed defense strategy that consists in an information advantage of the defender over the attacker, the attacker has a limited access to the intermediate results and does not know the used secret keys. Therefore, the attacker is not able to attack the proposed system in a *white-box* manner and to create directly the gradient-based adversarial examples. In this respect, the efficiency of the proposed multi-channel architecture with the diversification and randomization by the key-based sign flipping in the DCT domain against the adversarial attacks is tested for three scenarios:

1. *Gray-box* transferability attacks from a single-channel model to a multi-channel model tested on (i) the C&W attack [63] with the constraints on *ℓ*_2_, *ℓ*_0_, and *ℓ*_*∞*_ norms and (ii) the PGD attack [32].
2. *Gray-box* transferability attacks from a multi-channel model to a multi-channel model under different keys tested on the *OnePixel* attack [68] with perturbation in 1, 3, and 5 pixels.
3. *Black-box* direct attacks tested on the *OnePixel* attack [68] with perturbation in 1, 3, and 5 pixels.

**Fig. 10:**
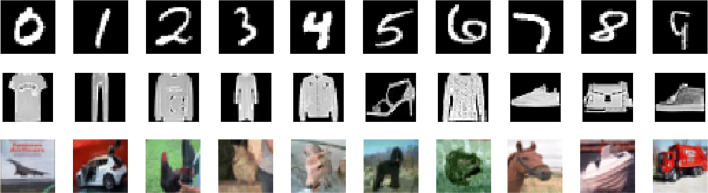
Examples of original images from each class from MNIST (top line), Fashion-MNIST (middle line), and CIFAR-10 (bottom line) data sets

**Fig. 11:**
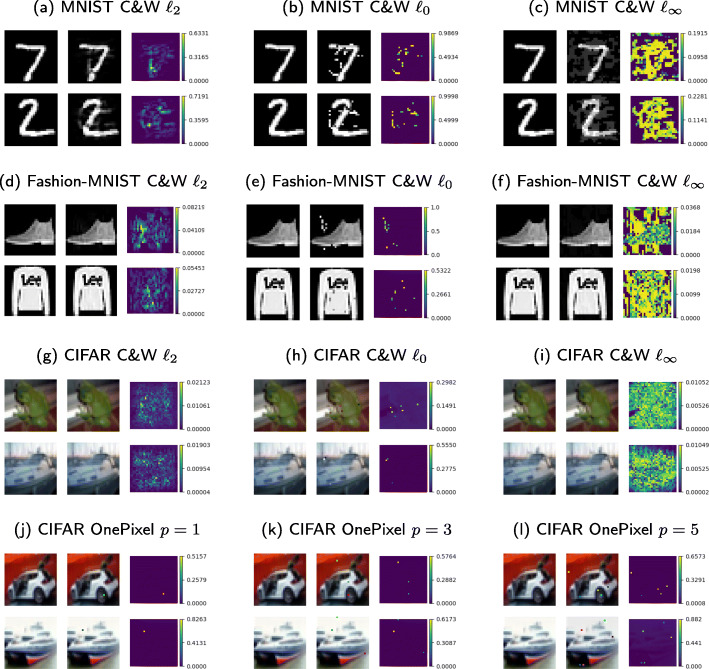
Adversarial examples: (left) original image ***x***, (middle) adversarial example ***x***^*adv*^, and (right) absolute value of the adversarial perturbation ***ε*** computed as |***ε***|=|***x***−***x***^*advv*^|

The experiments are performed on the MNIST [73], Fashion-MNIST [74], and CIFAR-10 data sets [75]. The MNIST set of handwritten digits contains 10 classes, 60,000 training and 10,000 test gray-scale images of the size 28×28. The Fashion-MNIST set has 10 classes, 60,000 training and 10,000 test gray-scale images of the size 28×28. The CIFAR-10 consists of 60,000 color images of size 32×32 (50,000 train and 10,000 test) with 10 classes. Examples of images from each data set are illustrated in Fig. 10. Due to the fact that the attack generation process is sufficiently slow for all considered attacks, the experimental results are obtained on the first 1000 test samples. The examples of the attacked images are given in Fig. 11.

The goal of experimental validation is to confirm whether the successful adversarial attacks can trick the proposed defense mechanism.

### Technical details

In this section, the technical details about the practical implementation of used attacks are presented. To ensure a reproducible research, the complete code is available at https://github.com/taranO/multi-channel-KDA.

#### C&W attack

For the fair comparison, the gradient-based C&W attack is tested on the classifiers with the architecture identical to those tested in [63]. The implementation was done in TensorFlow. For the training, the SGD was used with a learning rate 1e −2 and weight decay 1e −6. The “attacked vanilla” and “transferability vanilla” models were trained during 50 epochs (after 50th epoch, the saturation was observed) with a batch size equals to 128. In the multi-channel model, each classifier was trained during 100 epochs using Adam optimizer with a learning rate 1e −3, weight decay 1e −6, and batch size 64. For the *ℓ*_2_ attack, the learning rate was 1e −2, confidence 0, maximum number of iterations 1000 with early stopping if the gradient descent gets stuck, and the minimum and maximum pixel values equal to −0.5 and 0.5 correspondingly. For the *ℓ*_0_ and *ℓ*_*∞*_ attacks, the constant factor was 2, and the rest of the used parameter was the same as in case of *ℓ*_2_ attack.

#### OnePixel attack

The VGG16 [76] and ResNet18 [3] vanilla models were trained during 100 epochs with learning rate 1e −3, weight decay 5e −4, and batch size 128. For the VGG16, the SGD was used. In case of the ResNet18, the Adam was used. In multi-channel system for each classifier, the same corresponding parameters were used and the implementation was done in Pytorch.

#### PGD attack

The PGD attack was used to attack the VGG16 and ResNet18 models. The Pytorh implementation of PGD attack from the FoolBox library^1^ was used with the next parameters: *α* equals to 0.5, step size 0.01, and 100 iterations.

### Empirical results and discussion

Accordingly to the scenarios presented in Section 5.1, for each scenario, we provide the detailed explanation of (i) what kind of assumptions is done, (ii) what kind of knowledge is available to the attacker, and (iii) the obtained results.

#### Gray-box transferability: from a single-channel to a multi-channel

The results obtained for the *gray-box* transferability of the adversarial examples from a single-channel model to a multi-channel model are given in Table 1 for the C&W attack with the constraints on *ℓ*_2_, *ℓ*_0_ and *ℓ*_*∞*_ norms and in Table 2 for the PGD attack.

The architecture for a single-channel DNN classifier (that we named vanilla) was chosen and made known to the attacker. The attacker has also an access to the same training data set as the defender. The attacker trains his single-channel vanilla classifier and produces the adversarial examples against his system. The results of this attack are shown in the “Attacked vanilla” column of Tables 1 and 2. It is easy to see that the C&W attacks are very efficient against unprotected system. At the same time, Table 2 demonstrates that the PGD attack is less efficient.

The defender trains the same single-channel architecture using the same training data set but with different initialization of model’s parameters. The results of transferability of adversarial examples to the defender’s single-channel classifier are shown in the “Transferability vanilla” column of Tables 1 and 2. In contrast to the claimed transferability, our results clearly demonstrate the low efficiency of the proposed attacks even without any special defense mechanisms for the MNIST, Fashion-MNIST, and CIFAR-10 data sets.

The transferabillity of the same adversarial examples to the proposed multi-channel architecture produces the results reported in the “Transfereability KDA” column of Tables 1 and 2. The obtained results show that the increase of the number of channels leads to the decrease of classification error. More particularly, from Table 1, it is easy to see that in case of the CIFAR-10 data set that presents a particular interest for us as a data set with natural images, the classification error under the *C&Wℓ*_2_ and *C&Wℓ*_*∞*_ attacks is the same as in the case of the vanilla classifier on the original non-attacked data. In case of *C&Wℓ*_0_ attack, there is only about 2% of attack success. The similar situation can be observed for the the PGD attack given in Table 2. In the case of MNIST and Fashion-MNIST data sets, the *C&Wℓ*_2_ and *C&Wℓ*_*∞*_ produce about 1–3% of successful attacks while for the *C&Wℓ*_0_ this value is slightly higher and is about 2.5–5.5%. This is related to a high sparsity of the original images that, generally speaking, is not frequent for the natural images.

From the obtained results, one can conclude that the multi-channel model demonstrates the ability to be robust to the adversarial examples generated for the single-channel model with the same architecture of DNN classifier and the ability to improve the classification accuracy on both the non-attacked original and attacked data.

#### Gray-box transferability: from a multi-channel to a multi-channel

The results obtained for the *gray-box* transferability of the adversarial examples from one multi-channel model to another multi-channel model under different keys are given in Table 3 for the *OnePixel* attack with perturbation in 1, 3, and 5 pixels.

The architecture for a multi-channel model with the proposed defense strategy was made known to the attacker. The attacker has also access to the same training data set as the defender. The attacker does not know only the secret keys of the defender. Therefore he trains his multi-channel classifier under a selected set of keys and produces the adversarial examples against his system. The defender, in his turn, trains the similar system under different keys and different initialization of model’s parameters that remain secret. From the results reported in Table 3, it is easy to see that the success of attack does not exceed 0.5*%* compared to the classification accuracy on the original non-attacked data (rows “Original”). Moreover, as it is also observed in Tables 1 and 2, the increase of the number of channels in multi-channel model leads to the increase of classification accuracy both on the non-attacked original and attacked data.

#### Black-box direct attack

The results obtained for the direct attacks to a single-channel and a multi-channel models in the *black-box* scenario are shown in Table 4. The row “Original” corresponds to the use of non-attacked original data.

For a single-channel and a multi-channel cases, the attacker does not have any knowledge about the classifiers’ architecture, about the number of channels, or about the used defense mechanisms. The attacker can only observe the predicted class for the given input. In this respect, the attacker tries to attack the classification models directly in a black-box way. The results obtained for the *OnePixel* attack with perturbation in 1, 3, and 5 pixels are shown in Table 4. From these results, it is easy to see that for the non-protected single-channel model (“Attacked vanilla”), such kind of attacks can be sufficiently efficient: in the case of VGG16 model, the classification error is about 60–80%, and in the case of ResNet18 model, it is about 35–60%. For both classifiers, the increase of the number of perturbed pixels (*p*) leads to the increase of classification error. At the same time, the use of the proposed defense mechanism based on the KDA allows to decrease the classification error to the level of classification on the non-attacked original data, or in other words, it practically diminished the effect of these attacks.

To summarize the above results, it should be pointed out that as it can be seen from Tables 1, 2, 3, and 4, the results obtained for the non-attacked original data demonstrate that the use of the proposed multi-channel architecture, in general, allows to improve the classification accuracy of vanilla classifier. This is quite remarkable by itself since it shows that the multi-channel processing with the aggregation does not degrade the performance due to the introduced key-based diversification in contrast to many defense strategies based on the idea of randomization. Finally, the results obtained on the adversarial examples demonstrate high robustness of the proposed KDA-based defense technique.

Next, we demonstrate the impact of several factors, such as the key-based aggregation on the classification accuracy and robustness to the adversarial attacks (Tables 5 and 6) and the level of adversarial distortion (Table 7).

**Table 7:** Adversarial distortion

Attack	Median *ℓ*_2_-norm	Mean *ℓ*_2_-norm
*MNIST*		
*C&Wℓ*_2_	5.28e −03	5.52e −03
*C&Wℓ*_0_	1.56e −02	1.61e −02
*C&Wℓ*_*∞*_	1.24e −02	1.29e −02
*Fashion-MNIST*		
*C&Wℓ*_2_	2.30e −04	5.31e −04
*C&Wℓ*_0_	4.35e −03	4.86e −03
*C&Wℓ*_*∞*_	4.43e −04	5.43e −04
*CIFAR-10*		
*C&Wℓ*_2_	7.80e −05	1.19e −04
*C&Wℓ*_0_	2.48e −03	4.55e −03
*C&Wℓ*_*∞*_	1.73e −04	2.13e −04
*ResNet18 (CIFAR-10)*		
*PGD*	1.00e −04	1.37e −04
Vanilla *OnePixelp*=1	5.25e −04	2.76e −03
Vanilla *OnePixelp*=3	1.24e −03	3.51e −03
Vanilla *OnePixelp*=5	1.86e −03	4.18e −03
Multi-channel model *OnePixelp*=1	3.22e −04	2.42e −03
Multi-channel model *OnePixelp*=3	1.33e −03	3.61e −03
Multi-channel model *OnePixelp*=5	2.05e −03	4.33e −03
*VGG16 (CIFAR-10)*		
*PGD*	9.99e −05	1.50e −04
Vanilla *OnePixelp*=1	5.86e −04	2.78e −03
Vanilla *OnePixelp*=3	1.37e −03	3.69e −03
Vanilla *OnePixelp*=5	2.02e −03	4.26e −03
Multi-channel model *OnePixelp*=1	3.43e −04	2.25e −03
Multi-channel model *OnePixelp*=3	1.27e −03	3.64e −03
Multi-channel model *OnePixelp*=5	1.91e −03	4.28e −03

#### Key-based aggregation

Additionally to the multi-channel system with the fixed channels for aggregation shown in Fig. 9 and its results demonstrated in Tables 1, 3, and 4, the similar system has been investigated for the case when the channels for the aggregation were chosen based on a random key. The results averaged over 10 runs are given in Tables 2, 5, and 6. Comparing the results for the KDA presented, for example, in Tables 1 and 5, one can notice a small degradation of performance under the random selection of channels for the aggregation in Table 5. This is due to the fact that the sub-bands chosen for the randomization in the setup of Table 1 always correspond to the three main sub-bands representing V, H, and D sub-bands, whereas the sub-bands representing channels in the setup of Table 5 were chosen at random. This discrepancy decreases with the increase of the number of aggregated channels.

In summary, one can conclude that the obtained results indicate that the proposed KDA-based defense strategy demonstrates a high robustness to the transferability attacks in the *gray-box* scenarios as well as to the direct *black-box* attacks. Moreover, it allows to improve the classification accuracy of the vanilla classifiers. Finally, it should be pointed out that, in general, the increase of the number of classification channels and *data-independent processing*$\mathbb {P}_{ij}$ leads to improving the classification accuracy. However, a trade-off between the further decrease of the classification error and the increase of the complexity of the algorithm should be carefully addressed that goes beyond the scope of this paper.

#### Adversarial distortions

The efficiency of the proposed defense strategy with increased adversarial distortions in terms of amplitude value of the adversarial noise and its behavior are investigated in the gray-box scenario presented in Table 1. The amplitude of the adversarial noise increases from *ℓ*_2_ to *ℓ*_*∞*_ and *ℓ*_0_. Figure 11 shows several examples of adversarial noise with indicated noise amplitude. The median and mean *ℓ*_2_-norm of adversarial perturbation are given in Table 7. In all cases, the same trained model has been evaluated. The efficiency of the proposed defense strategy with the increase of adversarial distortions in terms of number of distorted pixels and its behavior are investigated in *black-box* scenario illustrated in Table 4, where *p*=1,... *p*=5 indicate the increase of the number of distorted pixels. The corresponding adversarial noise amplitudes are given in Table 7. In all cases, the proposed KDA-based defense strategy successfully resists to the adversarial distortion of different levels.

## Conclusions

In this paper, we address a problem of DNN classifiers’ protection against adversarial attacks in *gray-* and *black*-box scenarios. We propose the key-based randomized diversification mechanism as a defense strategy in the multi-channel architecture with the aggregation of classifiers’ scores. The randomized transform is a secret key-based randomization in a defined domain. The goal of this randomization is to prevent the gradient back propagation or use of “bypass” systems by the attacker. It is also important to remark that the proposed approach is “compliant” with the cryptographic principles when the defender has an information advantage over the attacker expressed via the knowledge of the secret key shared between the training and test stages. We evaluate the efficiency of the proposed defense and the performance of several variations of the considered architecture on three standard data sets against a number of known state-of-the-art attacks. The numerical results demonstrate the robustness of the proposed defense mechanism against (i) *gray-box* transferability attacks from a single-channel model to a multi-channel model under assumption that the attacker uses only the knowledge about the single-channel model architecture, (ii) *gray-box* transferability attacks from a multi-channel model to a multi-channel model trained under different keys assuming that the attacker has full knowledge about the multi-channel model architecture and used defense strategy except the defenders’ secret keys, and (iii) *black-box* direct attacks under assumption that the attacker has no knowledge about the model architecture or defense mechanisms. In all scenarios, as a worst case, we assume that the attacker uses the same data set as the defender. Additionally, the obtained results show that using the multi-channel architecture with the following aggregation stabilizes the results and increases the classification accuracy on the attacked and non-attacked original data samples.

For the future work, we aim at investigating in details the security aspects of the proposed KDA algorithm. It looks very interesting to obtain estimates and bounds on the attacker complexity attempting at learning the introduced randomization or bypassing it by some dedicated structures. It is also important to investigate the impact of number of training examples jointly with the randomization in terms of comparison of entropy of training data set versus needed entropy of randomization. Finally, it is important to extend the aggregation mechanism to more complex learnable strategies instead of used summation.

## Data Availability

The authors worked with publicly available databases [73–75].

## Abbreviations

DNN: Deep neural networks
CNN: Convilutional neural networks
KDA: Key-based diversified aggregation

## Footnotes

1 https://foolbox.readthedocs.io/en/stable/index.html
